# The relationship between career planning clarity and learning engagement among university students: the chain mediating role of self-efficacy and learning motivation

**DOI:** 10.3389/fpsyg.2026.1791804

**Published:** 2026-04-10

**Authors:** Jingjing Wang, Xue Xu, Shufen Lan, Wenzhe Huang

**Affiliations:** 1 School of Education Science, Hanshan Normal University, Chaozhou, China; 2 School of Arts and Sports, Shantou Preschool Education College in Guangdong, Shantou, China

**Keywords:** career planning clarity, learning engagement, learning motivation, self-efficacy, structural equation modeling

## Abstract

**Objective:**

This study aims to examine the impact of career planning clarity on learning engagement among university students, and to investigate the chain mediating roles of self-efficacy and learning motivation in this relationship.

**Methods:**

A questionnaire survey was conducted among 825 university students using the Career Planning Scale, the General Self-Efficacy Scale, and the Learning Motivation Scale.

**Results:**

(1) Career planning clarity, self-efficacy, learning motivation, and learning engagement were significantly positively correlated with each other; (2) Career planning clarity positively predicted learning engagement; (3) Both self-efficacy and learning motivation independently mediated the relationship between career planning clarity and learning engagement. Furthermore, the chain mediating effect of self-efficacy and learning motivation was also significant.

## 1 Introduction

In recent years, against the dual backdrop of the massification of higher education and an increasingly competitive job market, the learning quality and career development potential of university students have become key indicators for evaluating the effectiveness of higher education institutions. Learning engagement, a core variable reflecting students’ degree of participation, concentration, and Learning engagement, a core variable reflecting students’ degree of participation, concentration, and persistence in academic activities, not only directly influences dimensions of learning quality such as knowledge acquisition, skill development, and comprehensive competency enhancement but is also closely related to the overall quality of higher education and its talent cultivation structure (Wu and Yang, 2023). Traditionally, research on learning engagement has been predominantly situated within conventional classroom settings. However, with the emergence and proliferation of digital and blended learning environments in recent years, the forms of student learning have become increasingly diverse (Li and Xue, 2023; Alemayehu and Chen, 2021), making it imperative to continue investigating the mechanisms that sustain learning engagement across varied educational contexts. However, many current students struggle with issues such as vague career goals, unclear developmental pathways, and weak learning motivation, leading to learning engagement characterized by passivity, superficiality, and low persistence (Lei et al., 2024). Within this context, exploring how to effectively enhance university students’ learning engagement and understand its underlying mechanisms has become a significant research topic in the fields of higher education management and student career development.

Career planning clarity is a crucial psychological resource influencing students’ learning engagement, reflecting the degree of an individual’s cognitive clarity regarding career goals, pathways to achieve them, and the requisite competencies (Strauss et al., 2012). Its association with learning engagement has been preliminarily validated (Wu, 2018). Nevertheless, existing research exhibits notable limitations. On one hand, most studies treat career planning clarity merely as an exogenous variable, focusing only on its superficial correlation with learning engagement while neglecting the mediating role of internal psychological mechanisms. On the other hand, the potential dynamic interplay between career planning clarity and other psychological variables, such as self-efficacy and learning motivation, remains insufficiently explored, making it difficult to elucidate the mechanisms through which cognitive and motivational factors operate within the process of career planning influencing learning engagement. According to Social Cognitive Career Theory (Lent et al., 1994), individuals’ career development behaviors are significantly influenced by cognitive-motivational factors, wherein self-efficacy is considered a core cognitive variable playing a key role in goal setting and behavioral persistence (Bandura, 1977). In educational psychology, learning motivation is widely recognized as a proximal predictor of learning engagement (Alemayehu and Chen, 2021). Therefore, this study focuses on the relationship between university students’ career planning clarity and learning engagement, while simultaneously examining the mediating roles of self-efficacy and learning motivation. By adopting an integrated perspective combining career development and cognitive motivation theories, this research aims to provide a theoretical basis and practical insights for higher education institutions seeking to enhance the quality of student learning engagement.

### 1.1 Literature review and research hypothesis

Career planning clarity refers to the process through which individuals, after comprehensively understanding their own characteristics and the external environment, formulate and execute corresponding plans based on their developmental goals (Yan, 2013). Learning engagement denotes the intensity of behavioral involvement, the quality of emotional experience, and the cognitive strategies employed by students in learning activities (Heo et al., 2022). Existing research indicates that clarity in career planning goals can positively predict learning status. Specifically, individuals with clearer career plans tend to exhibit stronger self-discipline, consequently engaging in more learning behaviors (Wang, 2017) and demonstrating higher levels of learning participation (Strauss et al., 2012). Based on this, the following hypothesis is proposed:

*H1*: Career planning clarity positively influences learning engagement.

Self-efficacy refers to an individual’s subjective judgment of their capability to perform a specific action or task and to achieve desired outcomes, a judgment formed based on past experiences of success and failure (Bandura, 1977). According to Social Cognitive Career Theory, the process of career planning involves the continual reinforcement of self-efficacy. When individuals have clearer career plans, their self-efficacy tends to be higher, leading to greater investment required to achieve those plans (Lee et al., 2015). Higher self-efficacy positively influences persistence, goal setting, and self-regulation, thereby promoting learning engagement (Trowler et al., 2022). Cross-sectional studies have found that individuals with high self-efficacy are more likely to perceive learning tasks or difficulties as challenges and actively engage with them, resulting in more positive and effective learning engagement (Cai and Jia, 2020). Concurrently, longitudinal research has shown that high school students with clear future career plans experience increased self-efficacy upon reaching checkpoints toward their goals, thereby fostering closer engagement with their academics (Plasman, 2018). Accordingly, the following hypothesis is proposed:

*H2*: Self-efficacy mediates the relationship between career planning clarity and learning engagement.

Learning motivation refers to the psychological drive or internal force that activates or sustains an individual’s learning activities and directs learning behavior toward specific goals. It plays a crucial role in regulating and controlling student learning behaviors and guiding deep learning (Huang and Cheng, 2024). Existing research has found that clear career goals positively influence an individual’s motivation to engage in learning behaviors (Li et al., 2019). Furthermore, according to motivation theory, when facing a difficult task, stronger learning motivation enables individuals to respond more positively and persist (Kanfer, 1990). Empirical studies support this view; for instance, research on Chinese vocational college students (Guo et al., 2025) found that career planning clarity enhances learning engagement by strengthening individual learning motivation. Based on this, the following hypothesis is proposed:

*H3*: Learning motivation mediates the relationship between career planning clarity and learning engagement.

The Basic Psychological Needs Theory, a sub-theory of Self-Determination Theory, posits that the transition between internal and external motivation is a natural process, but not unconditional. The need for competence is one of the basic psychological needs facilitating this motivational shift, and the need for competence is synonymous with self-efficacy (Ye et al., 2016), meaning self-efficacy positively predicts learning motivation. Existing research supports this relationship. For example, a study on physical education among Chinese university students found that an individual’s self-efficacy influences their level of learning motivation, and higher motivation levels subsequently promote learning engagement (Huang and Cheng, 2024). Synthesizing the above theoretical analysis and previous research suggests that university students’ career planning might positively influence learning engagement by enhancing self-efficacy, which in turn stimulates learning motivation. Accordingly, the following hypothesis is proposed:

*H4*: Self-efficacy and learning motivation play a chain mediating role in the relationship between career planning clarity and learning engagement.

**Figure 1:**
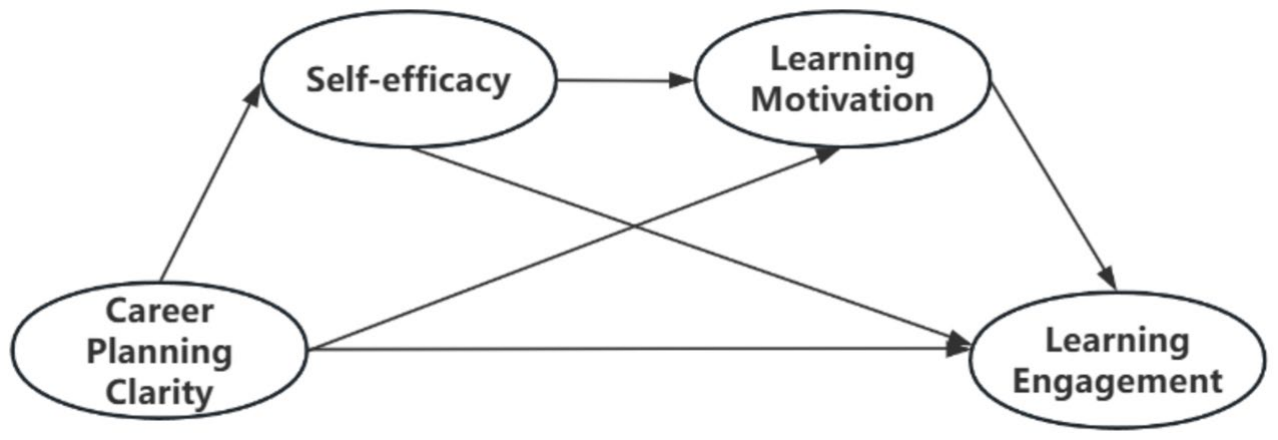
The hypothesized model of career planning clarity affecting learning engagement.

In summary, this study takes career planning clarity as the starting point and, drawing on the perspectives of self-efficacy and learning motivation, constructs a chain mediation model (Figure 1) to reveal the mechanism through which university students’ career planning clarity affects learning engagement. The findings aim to provide a theoretical basis for enhancing student learning engagement and contribute to enriching related research.

## 2 Research methods

### 2.1 Participants

Undergraduate students from a normal university in Guangdong Province, China, were recruited as participants. A total of 1,031 questionnaires were collected via an online survey. After excluding questionnaires with excessively short response times and patterned responses, 825 valid questionnaires were retained, yielding an effective response rate of 80.02%. Among the respondents, 259 were male (31.40%) and 566 were female (68.60%); 508 majored in Arts/Humanities (61.60%) and 317 in Sciences (38.40%). Regarding academic year, 169 were freshmen (20.50%), 176 were sophomores (21.30%), 188 were juniors (22.80%), and 292 were seniors (35.40%).

### 2.2 Research instruments

#### 2.2.1 Career planning clarity

The Career Planning Scale for University Students, developed by Liu (2017) based on career development theory, was used. This scale has demonstrated good reliability and validity in previous studies. It comprises six dimensions: Career Awareness (e.g., “I believe career planning is important to me”), Self-Exploration (e.g., “I am clear about my interests and hobbies”), Environmental Exploration (e.g., “I actively seek information about the employment prospects of my desired industry”), Career Decision-Making (e.g., “I am unclear about my career direction” - reverse scored), Career Action (e.g., “I participate in club activities to develop the qualities and skills required for my desired career”), and Career Adjustment (e.g., “In the past year, I have changed my perception of myself”). The scale consists of 19 items, such as “I have set career goals for one year after graduation.” Responses were rated on a 5-point Likert scale ranging from 1 (Strongly Disagree) to 5 (Strongly Agree), with higher total scores indicating greater career planning clarity. In this study, the overall Cronbach’s *α* coefficient for the scale was 0.90. Confirmatory Factor Analysis (CFA) indicated acceptable model fit indices: χ^2^/df = 3.32, NFI = 0.91, CFI = 0.94, IFI = 0.94, RMSEA = 0.05.

#### 2.2.2 Self-efficacy

The General Self-Efficacy Scale (Schwarzer and Jerusalem, 1995) was used to assess individuals’ general sense of perceived self-efficacy. It contains 10 items (e.g., “I can always manage to solve difficult problems if I try hard enough”). Respondents rated themselves on a 4-point Likert scale (1 = Strongly Disagree, 4 = Strongly Agree). Higher scores indicate higher levels of self-efficacy. In this study, the scale’s overall Cronbach’s *α* coefficient was 0.79. CFA results showed acceptable fit indices: χ^2^/df = 2.62, NFI = 0.84, CFI = 0.89, IFI = 0.89, RMSEA = 0.06.

#### 2.2.3 Learning motivation

The Learning Motivation Scale developed by Yu et al. (1987) was employed. This scale consists of two dimensions: Self-Oriented Achievement Motivation (intrinsic motivation) and Socially-Oriented Achievement Motivation (extrinsic motivation). It contains 13 items (e.g., “I study hard to receive praise and encouragement”). Responses were given on a 5-point Likert scale. Higher total scores indicate stronger learning motivation. The scale included two reverse-scored items. In this study, the overall Cronbach’s *α* coefficient for the scale was 0.85. CFA results indicated acceptable model fit: χ^2^/df = 4.02, NFI = 0.86, CFI = 0.88, IFI = 0.88, RMSEA = 0.08.

#### 2.2.4 Learning engagement

The Learning Engagement Questionnaire compiled by Liao (2011) was used. This questionnaire includes three dimensions: Behavioral Engagement, Cognitive Engagement, and Emotional Engagement. It comprises 18 items (e.g., “I often check my learning progress against my learning goals”). Higher scores indicate a higher level of learning engagement. In this study, the overall Cronbach’s α coefficient for the questionnaire was 0.91. CFA results showed acceptable fit indices: χ^2^/df = 4.83, NFI = 0.89, CFI = 0.91, IFI = 0.91, RMSEA = 0.07.

### 2.3 Data analysis

Data were analyzed using SPSS 27.0 and the PROCESS macro for descriptive statistics, common method bias test, correlation analysis, and chain mediation analysis. Amos 23.0 was used to conduct Confirmatory Factor Analysis (CFA) for the scales.

## 3 Results

### 3.1 Common method bias test

Since this study relied solely on questionnaire surveys, common method bias (CMB) may be a concern. To minimize the potential impact of CMB on the accuracy of the findings and to ensure the reliability of the model analysis, both procedural and statistical approaches were employed. Procedurally, the order of items was carefully arranged, the measurement purpose of each item was concealed, and participants were informed that the survey data would be used solely for academic research. All responses were anonymous, and participants were assured that their teachers would not have access to their answers, encouraging honest responses based on their actual circumstances and thereby reducing social desirability bias. Statistically, Harman’s single-factor test was conducted, and the results indicated that the first factor accounted for 30.63% of the total variance, below the 40% threshold, suggesting that common method bias was not a serious issue in this study.

### 3.2 Descriptive statistical analysis of variables

The results of the descriptive statistics and correlation analysis among the variables are presented in Table 1. As shown in the table, significant positive correlations were found among all variable dimensions.

**Table 1:** Descriptive statistics and correlation analysis among variables.

Variables	*M*	*SD*	Career planning clarity	Self -efficacy	Learning motivation	Learning engagement
Career planning clarity	3.22	0.72	1			
Self-efficacy	2.73	0.55	0.65^**^	1		
Learning motivation	3.11	0.69	0.82^**^	0.64^**^	1	
Learning engagement	3.18	0.74	0.82^**^	0.66^**^	0.80^**^	1

^**^p < 0. 01.

### 3.3 Regression analysis

#### 3.3.1 Direct effect analysis

Regression analysis was conducted using the SPSS macro PROCESS developed by Hayes (2013). As shown in Table 2, career planning clarity positively predicted learning engagement (*β* = 0.43, *p* < 0.001). Concurrently, career planning clarity also positively predicted self-efficacy (*β* = 0.50, *p* < 0.001) and learning motivation (*β* = 0.67, *p* < 0.001). Both self-efficacy and learning motivation significantly predicted learning engagement (*β* = 0.22, *p* < 0.001; *β* = 0.37, *p* < 0.001, respectively). The overall regression model was significant, R^2^ = 0.82, *F* (1, 823) = 1664.13, *p* < 0.001.

**Table 2:** Regression analysis of variables in the model.

Outcome variable	Predictor variable	*R*	*R* ^2^	*F*	β	*t*
Self-efficacy	Career planning clarity	0.65	0.42	603.63^***^	0.50	24.57^***^
Learning motivation	Career planning clarity	0.83	0.70	936.42^***^	0.67	27.59^***^
	Self-efficacy				0.24	7.38^***^
Learning engagement	Career planning clarity	0.86	0.74	764.14^***^	0.43	12.99^***^
	Self-efficacy				0.22	6.66^***^
	Learning motivation				0.37	10.77^***^

^***^p < 0. 001.

#### 3.3.2 Indirect effect analysis

**Figure 2:**
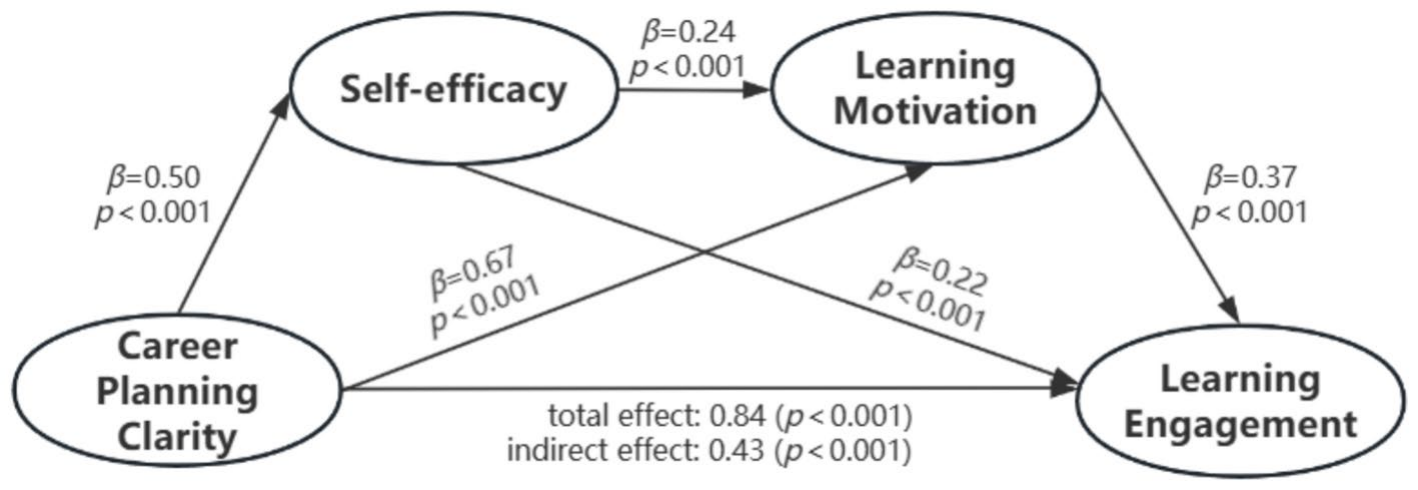
The chain mediating effect of self-efficacy and learning motivation.

The mediation effects were tested using the Bootstrap sampling method. The results, presented in Table 3 and Figure 2, indicated that all path coefficients in the model were significant. The total indirect effect was 0.40, with a 95% confidence interval (CI) of [0.34, 0.46]. Specifically, the indirect effect via self-efficacy was 0.11 (95% CI [0.07, 0.14]), indicating a significant mediating role of self-efficacy. The indirect effect via learning motivation was 0.25 (95% CI [0.20, 0.30]), confirming its significant mediating role. The chain mediating effect of self-efficacy and learning motivation in the relationship between career planning clarity and learning engagement was 0.04 (95% CI [0.02, 0.06]), demonstrating a significant chain mediation effect.

**Table 3:** Test of the chain mediating effect of self-efficacy and learning motivation.

Indirect type	Path relationship	*β*	Boot SE	95% CI	Proportion mediated
Total indirect		0.40	0.03	[0.34,0.46]	47.62%
Indirect 1	CCP → SE → LM	0.11	0.02	[0.07,0.14]	13.10%
Indirect 2	CCP → LM → LE	0.25	0.03	[0.20,0.30]	29.76%
Indirect 3	CCP → SE → LM → LE	0.04	0.01	[0.03,0.06]	4.76%

CCP = career planning clarity, SE = self-efficacy, LM = learning motivation, LE = learning engagement.

## 4 Discussion

### 4.1 The influence of career planning clarity on learning engagement

The results of this study indicate that university students’ career planning clarity positively predicts their learning engagement, supporting Hypothesis H1. This finding aligns with previous research (Xie and Yin, 2021). When students establish clear career goals through self-exploration and environmental assessment, their daily learning activities are no longer perceived as isolated or abstract tasks but are viewed as essential steps and capital for achieving future career aspirations. This reconceptualization of the value of learning can effectively stimulate students’ proactive engagement, making them more willing to invest sustained effort (behavioral engagement), experience immersive enjoyment (emotional engagement), and employ flexible and efficient strategies (cognitive engagement), thereby demonstrating a higher level of overall learning engagement. Therefore, for university students, exploring the external career environment and their own characteristics to clarify career plans is a crucial task, holding significant importance for future personal development and for enhancing their learning participation and persistence.

### 4.2 The separate mediating roles of self-efficacy and learning motivation

The results demonstrate that career planning clarity influences learning engagement through self-efficacy, supporting Hypothesis H2. This suggests that self-efficacy is an important internal mechanism through which career planning clarity affects university students’ learning engagement, a finding consistent with prior studies (Su, 2021). Effective career planning can significantly enhance students’ self-efficacy in both academic and future professional domains. High levels of academic self-efficacy, in turn, directly drive deeper learning engagement (Getenet et al., 2024). Students with high self-efficacy firmly believe in their ability to master knowledge and successfully handle academic challenges. Consequently, they are more inclined to set challenging learning goals, demonstrate greater resilience and persistence in the face of difficulties, and utilize deep learning strategies more frequently (Honicke and Broadbent, 2016)—all of which are core manifestations of learning engagement.

This study also found that career planning clarity influences learning engagement through learning motivation, confirming Hypothesis H3. This result is consistent with previous research (Ji and Wang, 2016; Gao, 2020). Self-Determination Theory (SDT) posits that the key to fostering sustained individual engagement lies in satisfying their need for autonomy, thereby stimulating their intrinsic motivation or highly internalized extrinsic motivation. Career planning, which emphasizes autonomous choices based on personal interests and values rather than external imposition, fulfills this basic psychological need essential for the internalization of motivation.

### 4.3 The chain mediating effect of self-efficacy and learning motivation

This study found that self-efficacy and learning motivation play a chain mediating role between career planning clarity and learning engagement, validating Hypothesis H4. This conclusion is largely consistent with previous research (Huang, 2018). The Motivation and Engagement Wheel (MEW) theory proposes that both self-efficacy and motivation are adaptive cognitions that positively influence learning engagement, an adaptive behavior (Martin, 2007). Corresponding research also indicates that self-efficacy is a key intrinsic driver for stimulating learning motivation; students with stronger self-beliefs in their capabilities tend to have stronger learning motivation, leading to more significant learning outcomes (Huang and Cheng, 2024). This finding suggests that the impact of career planning on learning engagement is not a simple direct causal relationship but rather a sequential cognitive-motivational process involving multiple psychological resources. It first helps students construct a clear future orientation and goal system. This system then enhances students’ “I can do it” belief (self-efficacy), which in turn strengthens their perception of the meaning behind learning and their drive (“why to learn”), ultimately translating these cognitive and motivational resources into the persistent behavioral manifestation of “actively learning” (learning engagement). In summary, the confirmation of this chain mediation model not only deepens our understanding of the value of career planning education but also provides a precise intervention pathway for enhancing academic ethos and talent cultivation quality in higher education institutions through “career education.”

### 4.4 Implications

Based on the empirical findings, this study proposes the following implications for higher education:

First, universities should systematically construct a career education system integrated throughout the entire talent cultivation process. By offering specialized career planning courses, conducting career interest assessments, organizing case studies, and providing practical experiences, institutions can help students comprehensively understand their own characteristics and the external occupational environment, thereby forming clear career development paths and achieving mutual reinforcement between the learning process and career planning.

Second, in the process of teaching and education, instructors should focus on stimulating students’ intrinsic learning motivation. Through workshops, individual counseling, opportunities for staged successful experiences, and role modeling, teachers can enhance students’ sense of competence and autonomy. Supportive verbal feedback regarding their abilities and skill mastery should be provided. According to Cognitive Evaluation Theory (Zhao et al., 2016), controlling external rewards may undermine intrinsic motivation, whereas feedback that supports autonomy and perceived competence helps strengthen it. Therefore, instructors should avoid overemphasizing controlled targets like credits and grades. Instead, they should strive to build a virtuous cycle of “career cognition → motivation/efficacy → learning engagement,” facilitating the shift from passive (“have to learn”) to active (“want to learn”) learning, thereby enhancing students’ learning quality and career development potential.

### 4.5 Limitations and future research directions

This study investigated the relationship between university students’ career planning clarity and learning engagement, further expanding related research. However, several limitations should be acknowledged and addressed in future studies: First, the cross-sectional design limits the ability to definitively establish causal relationships between the variables. Future research could employ longitudinal or cross-lagged designs to further examine and supplement these findings. Second, all data were collected via self-report measures, which may be subject to biases. Future studies could combine questionnaires with methods like interviews to obtain more comprehensive and objective results. Additionally, this study employed a general self-efficacy scale. Future research may further compare the differential effects of general self-efficacy and career-specific self-efficacy (e.g., career decision-making self-efficacy) within the model, which would help deepen the understanding of how different types of efficacy beliefs function in the relationship between career planning and learning engagement. Last, this study’s sample was drawn exclusively from universities in Guangdong Province, a region with a developed economy and competitive labor market, which may influence students’ career planning clarity. Additionally, factors such as peer relationships and socioeconomic status were not examined due to scope limitations. Future research could employ more rigorous designs to explore how career planning affects learning engagement across different peer contexts, family backgrounds, and regions with varying levels of economic development.

## 5 Conclusion

1. Career planning clarity, self-efficacy, learning motivation, and learning engagement were all significantly positively correlated with each other.
2. University students’ career planning clarity positively predicted their learning engagement.
3. Self-efficacy and learning motivation separately mediated the relationship between career planning clarity and learning engagement.
4. Self-efficacy and learning motivation played a chain mediating role between career planning clarity and learning engagement.

## Data Availability

The raw data supporting the conclusions of this article will be made available by the authors, without undue reservation.

## References

[ref1] Alemayehu L. Chen H. L. (2021). The influence of motivation on learning engagement: the mediating role of learning self-efficacy and self-monitoring in online learning environments. Interact. Learn. Environ. 31, 4605–4618. doi: 10.1080/10494820.2021.1977962

[ref2] Bandura A. (1977). Self-efficacy: toward a unifying theory of behavioral change. Psychol. Rev. 84, 191–215. doi: 10.1037/0033-295x.84.2.191, 847061

[ref3] Cai L. Jia X. J. (2020). The relationship between academic self-efficacy and online learning engagement: the chain mediating role of learning motivation and flow experience. Psychol. Behav. Res. 18, 805–811.

[ref4] Gao X. M. (2020). Characteristics of contemporary college students' learning motivation and its impact on academic achievement. Higher Educ. Exploration 1, 43–47.

[ref5] Getenet S. Cantle R. Redmond P. Albion P. (2024). Students’ digital technology attitude, literacy and self-efficacy and their effect on online learning engagement. Int. J. Educ. Technol. High. Educ. 21:3. doi: 10.1186/s41239-023-00437-y

[ref6] Guo T. Sun Q. Liang Y. Li D. Yan H. Wu W. (2025). Vocational students’ career-planning clarity and learning engagement: a moderated mediation model. Soc. Behav. Pers. 53, 1–13. doi: 10.2224/sbp.13380

[ref002] Hayes A. F. (2013). Introduction to mediation, moderation, and conditional process analysis: A regression-based approach. The Guilford Press.

[ref7] Heo H. Bonk C. J. Doo M. Y. (2022). Influences of depression, self-efficacy, and resource management on learning engagement in blended learning during COVID-19. Internet High. Educ. 54:100856. doi: 10.1016/j.iheduc.2022.100856, 35464172 PMC9013013

[ref8] Honicke T. Broadbent J. (2016). The influence of academic self-efficacy on academic performance: a systematic review. Educ. Res. Rev. 17, 63–84. doi: 10.1016/j.edurev.2015.11.002

[ref9] Huang R. Y. (2018). Exploring the Relationship between Career Planning, Self-Efficacy, and Learning Motivation among Ordinary high school Students (Doctoral Dissertation). Zhangzhou, China: Minnan Normal University.

[ref10] Huang W. Z. Cheng B. J. (2024). The impact of exercise self-efficacy on college students' technical learning engagement in physical education: the chain mediating role of physical education learning motivation and flow experience. Sports Sci. Res. 45, 58–65+77.

[ref11] Ji Y. Wang Y. S. (2016). The impact of learning motivation on college students' learning engagement: the mediating effect of interpersonal interaction. Higher Educ. Exploration 12, 23–28.

[ref12] Kanfer R. (1990). Motivation Theory and Industrial and Organizational Psychology. üPalo Alto, CA: Consulting Psychologists Press.

[ref14] Lee H. S. Flores L. Y. Navarro R. L. Kanagui-Munoz M. (2015). A longitudinal test of social cognitive career theory’s academic persistence model among Latino/a and white men and women engineering students. J. Vocat. Behav. 88, 95–103. doi: 10.1016/j.jvb.2015.02.003

[ref15] Lei H. Chen C. Luo L. (2024). The examination of the relationship between learning motivation and learning effectiveness: a mediation model of learning engagement. Humanit. Soc. Sci. Commun. 11, 1–11. doi: 10.1057/s41599-024-02666-6

[ref16] Lent R. W. Brown S. D. Hackett G. (1994). Toward a unifying social cognitive theory of career and academic interest, choice, and performance. J. Vocat. Behav. 45, 79–122. doi: 10.1006/jvbe.1994.1027

[ref17] Li H. Ngo H. Y. Cheung F. (2019). Linking protean career orientation and career decidedness: the mediating role of career decision self-efficacy. J. Vocat. Behav. 115:103322. doi: 10.1016/j.jvb.2019.103322

[ref18] Li J. Xue E. (2023). Dynamic interaction between student learning behaviour and learning environment: meta-analysis of student engagement and its influencing factors. Behav. Sci. 13:59. doi: 10.3390/bs13010059, 36661631 PMC9855184

[ref19] Liao Y. G. (2011). Development and current situation investigation of college students' learning engagement questionnaire. J. Jimei Univ. 2, 39–44.

[ref20] Liu J. Z. (2017). Research on the impact of Career Planning on College Students' Career Maturity (Doctoral Dissertation). Chengdu, China: Southwest Jiaotong University.

[ref21] Martin A. J. (2007). Examining a multidimensional model of student motivation and engagement using a construct validation approach. Br. J. Educ. Psychol. 77, 413–440. doi: 10.1348/000709906X118036, 17504555

[ref22] Plasman J. S. (2018). Career/education plans and student engagement in secondary school. Am. J. Educ. 124, 217–246. doi: 10.1086/695608

[ref23] Schwarzer R. Jerusalem M. (1995). “Generalized self-efficacy scale,” in Measures in Health Psychology: A User’s Portfolio. Causal and Control Beliefs, eds. Weinman J. Wright S. Johnston M. (Windsor, UK: NFER-Nelson), 35–37.

[ref24] Strauss K. Griffin M. A. Parker S. K. (2012). Future work selves: How salient hoped-for identities motivate proactive career behaviors. J. Appl. Psychol. 97, 580–598. doi: 10.1037/a0026423, 22122111

[ref25] Su P. P. (2021). Research on the Relationship between Achievement goal Orientation, Self-Efficacy, and academic Achievement among College Students (Master's thesis). Nanjing, China: Nanjing University of Posts and Telecommunications.

[ref26] Trowler V. Allan R. L. Bryk J. Din R. R. (2022). Pathways to student engagement: beyond triggers and mechanisms at the engagement interface. High. Educ. 84, 761–777. doi: 10.1007/s10734-021-00798-1

[ref27] Wang J. W. (2017). A Survey and Research on the Correlation between Career goal Clarity and Learning status of Ordinary high school Students (Master's thesis). Shijiazhuang, PR: Hebei Normal University.

[ref29] Wu L. Q. (2018). Research on the impact of Career Planning on Learning Engagement of academic Master's Students in Sports Science (Master's thesis). Beijing, China: China University of Mining and Technology.

[ref30] Wu Y. C. Yang X. (2023). Research on influencing factors of learning engagement of new engineering college students: based on grounded theory analysis. Chinese Univ. Sci. Technol. 11, 58–64. doi: 10.16209/j.cnki.cust.2023.11.017

[ref32] Xie L. Yin J. (2021). The impact of career planning clarity on college students' learning engagement: a moderated mediation model. Psychol. Explor. 41, 437–442.

[ref33] Yan L. F. (2013). Analysis of youth career planning and problems: taking college students as an example. J. China Youth Univ. Polit. Sci. 32, 12–16. doi: 10.16034/j.cnki.10-1318/c.2013.01.013

[ref35] Ye B. J. Zheng Q. Liu L. L. Fang X. T. (2016). The impact of career exploration on college students' job search behavior: the mediating role of job search self-efficacy and the moderating role of emotion regulation. Psychol. Dev. Educ. 32, 691–697. doi: 10.16187/j.cnki.issn1001-4918.2016.06.07

[ref001] Yu A. B. Yang K. S. (1987). Social‑oriented and individual‑oriented achievement motivation: A conceptual and empirical analysis (in Chinese). Bulletin of the Institute of Ethnology, Academia Sinica, 64, 51–98.

[ref36] Zhao Y. M. Zhang Z. T. Liu N. Ding M. Z. (2016). A review of new developments in self-determination theory. J. Manage. 13, 1095–1104.

